# Transcribed Tc1-like transposons in salmonid fish

**DOI:** 10.1186/1471-2164-6-107

**Published:** 2005-08-12

**Authors:** Aleksei Krasnov, Heikki Koskinen, Sergey Afanasyev, Hannu Mölsä

**Affiliations:** 1 Institute of Applied Biotechnology, University of Kuopio, P.O.B. 1627, FIN-70211 Kuopio, Finland; 2 Sechenov Institute of Evolutionary Physiology and Biochemistry, M.Toreza av. 44, Petersburg, 194223, Russia

## Abstract

**Background:**

Mobile genetic elements comprise a substantial fraction of vertebrate genomes. These genes are considered to be deleterious, and in vertebrates they are usually inactive. High throughput sequencing of salmonid fish cDNA libraries has revealed a large number of transposons, which remain transcribed despite inactivation of translation. This article reports on the structure and potential role of these genes.

**Results:**

A search of EST showed the ratio of transcribed transposons in salmonid fish (i.e., 0.5% of all unique cDNA sequences) to be 2.4–32 times greater than in other vertebrate species, and 68% of these genes belonged to the Tc1-family of DNA transposons. A phylogenetic analysis of reading frames indicate repeated transposition of distantly related genes into the fish genome over protracted intervals of evolutionary time. Several copies of two new DNA transposons were cloned. These copies showed relatively little divergence (11.4% and 1.9%). The latter gene was transcribed at a high level in rainbow trout tissues, and was present in genomes of many phylogenetically remote fish species. A comparison of synonymous and non-synonymous divergence revealed remnants of divergent evolution in the younger gene, while the older gene evolved in a neutral mode. From a 1.2 MB fragment of genomic DNA, the salmonid genome contains approximately 10^5 ^Tc1-like sequences, the major fraction of which is not transcribed. Our microarray studies showed that transcription of rainbow trout transposons is activated by external stimuli, such as toxicity, stress and bacterial antigens. The expression profiles of Tc1-like transposons gave a strong correlation (r^2 ^= 0.63–0.88) with a group of genes implicated in defense response, signal transduction and regulation of transcription.

**Conclusion:**

Salmonid genomes contain a large quantity of transcribed mobile genetic elements. Divergent or neutral evolution within genomes and lateral transmission can account for the diversity and sustained persistence of Tc1-like transposons in lower vertebrates. A small part of transposons remain transcribed and their transcription is enhanced by responses to acute conditions.

## Background

A large fraction of repetitive sequences originate in eukaryotic genomes from mobile genetic elements (MGEs), which are grouped into 2 classes. Class I transposons require mRNA intermediates, whereas class II elements transpose directly as DNA. Tc1-like class II transposons, named after the founder gene in *Caenorhabditis elegans*, are probably the most widespread MGEs in nature, and are found in fungi, plant ciliates, nematodes, arthropods, fish, amphibians and mammals (reviewed in [1]). These genes contain a single reading frame that encodes for the enzyme transposase, which is flanked with terminal inverted repeating units. Transposition of class II MGEs is characterized by limited requirements for host cellular factors, which can account for their remarkable ability to undergo horizontal transfer across great taxonomic distances [2]. MGEs are regarded as parasitic genes, and proliferation is deleterious for the host. Therefore, transposition is commonly followed by inactivation. MGEs could play an important role in the evolution of teleost fish, and comprise a substantial fraction of their genome. Multiple copies of Tc1-like transposons were found in several fish species from different orders [3-6], however transcription of teleost Tc1-like genes has not been documented. Recent high-throughput sequencing of salmonid cDNA libraries has revealed surprisingly large number of transposon transcripts. Most if not all these sequences contain incapacitating mutations in the reading frames, and can be regarded as transcribed pseudogenes or null-alleles. At present, rainbow trout (*Oncorhynchus mykiss*) and Atlantic salmon (*Salmo salar*) TIGR Gene Indices [7] contain 50773 and 31341 unique cDNA sequences, respectively among which we found several hundreds MGE, Tc1-like genes being most abundant. This wealth of sequence information provides insight into the structure and evolution of transposons. We also cloned several copies of two rainbow trout Tc1-like genes with complete reading frames, which adds to understanding the transposon life cycle. Multiple gene expression analyses with high-density cDNA microarray indicate stimulation of rainbow trout transposons transcription in response to stress, toxicity and pathogens.

## Results

In order to search for transcribed transposons in salmonid fish, we compared the unique cDNA sequences from TIGR gene indices with 262 metazoan transposon proteins retrieved from Swissprot. Blastx found matches in 273 rainbow trout and 163 Atlantic salmon sequences at a cutoff value e < 10^-20 ^(Table 1). The ratio of transposons to all cDNA sequences in salmonids was 2.35–31.6 times greater than in other vertebrate species with available gene indices, and a large fraction (68.3%) showed similarity to 11 proteins of the Tc1 family. Tc1-like transcripts were found in the gene indices of 4 other teleost fish species and in the African clawed frog *Xenopus laevis*, but not in higher vertebrates. To estimate an approximate number of Tc1-like genes, 6 genomic clones of Atlantic salmon were analyzed, covering 1.2 MB [Genbank:AC148723, Genbank:AC149099, Genbank:AC148779, Genbank:AC148618, Genbank:AC148617 and AC148616], and a blastx search found 56 matches at a cutoff value of 10^-20^. The size of the haploid Atlantic salmon genome is equal to 3 billion base pairs. Assuming a relatively homogenous distribution of Tc1-transposons, about 140,000 copies can be expected, which is 3 orders of magnitude greater than the number of Tc1-like sequences in the salmonid fish gene indices. It is necessary to note that TIGR contigs are produced by automatic assembly of EST sequences that have at least 95% homology in overlaps of minimum units of 40 base pairs [8]. Therefore, transcripts of recently diverged transposon copies could be merged unless they were flanked by differing 5'- and 3'-untranslated sequences. The numbers of transcribed transposons can be greater than the number estimated by searching across gene indices, but it is likely that only a minor fraction of salmonid Tc1-like genes is active.

**Table 1:** Transcribed vertebrate transposons^1^.

**Species**	**Sequences**	**Transposons**	**Tc1-like**
Rainbow trout (*Oncorhynchus mykiss*)	50773	273 (0.538)	188
Atlantic salmon (*Salmo salar*)	31341	163 (0.52)	110
Medaka (*Oryzias latipes*)	26689	59 (0.221)	8
Zebrafish (*Danio rerio*)	93442	194 (0.208)	84
Killifish (*Fundulus heteroclitus*)	15538	3 (0.019)	3
Pufferfish (*Takifugu rubripes*)	11112	11 (0.099)	1
African clawed frog (*Xenopus laevis*)	77599	164 (0.211)	53
Chicken (*Gallus gallus*)	116777	20 (0.017)	0
Rat (*Rattus norvegicus*)	147056	310 (0.211)	0

^1^Unique cDNA sequences from TIGR Gene Indexes were searched across 262 metazoan transposons from Swissprot and 11 Tc1-like transposases using blastx at a cutoff value of e < 10^-20^. Percentages for all cDNA sequences are in parentheses.

**Figure 1:**
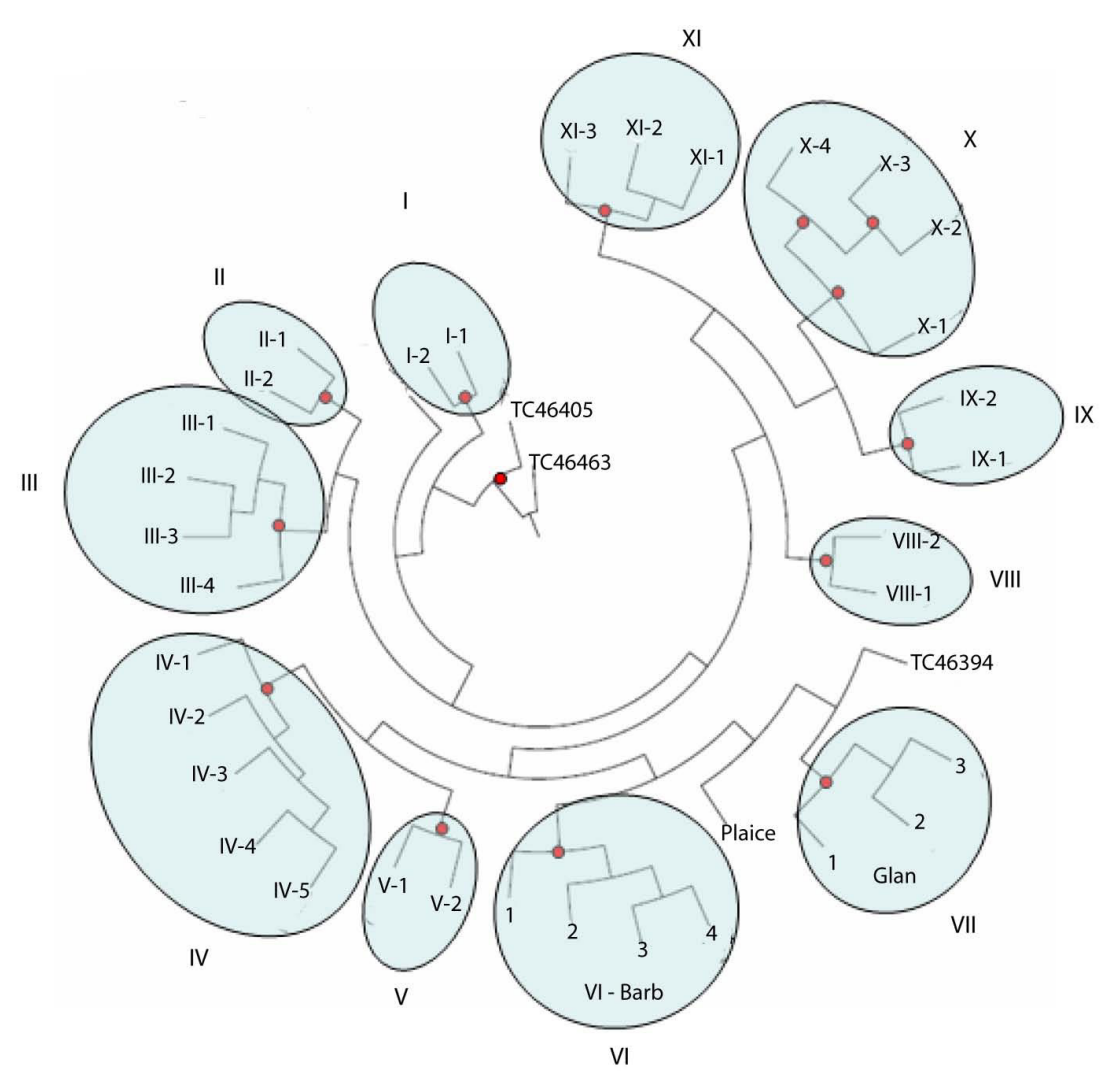
Structural relatedness of transcribed rainbow trout Tc1-like transposons. The ML tree is based on sequences encoding for at least 170 amino acids at the C-termini. The TIGR sequences are designated by the accession numbers, transposons Barb and Glan were identified in this study. Tree was produced using Dnaml (Phylip package), nodes with bootstrap values greater than 0.75 are indicated. Accession numbers of TIGR contigs in the clusters are: I-1 – BX884691; I-2 – TC46229; II-1 – TC52875; II-2 – TC46539; III-1 – TC46343; III-2 – TC46498; III-3 – TC46491; III-4 – CB488722; IV-1 – TC46455; IV-2 – CA377451; IV-3 – TC47500; IV-4 – TC47499; IV-5 – CA369142; V-1 – TC46391; V-2 – CA369399; VIII-1 – TC54663; VIII-2 – TC54666; IX-1 – CB488927; IX-2 – TC46493; X-1 – TC46521; X-2 – TC46197; X-3 – TC46383; X-4 – TC46308; XI-1 – CA361855; XI-2 – TC54683; XI-3 – CR368829.

Most Tc1-like sequences from the rainbow trout gene index contained incomplete reading frames. To analyse the structural relatedness of these genes, we used 38 fragments, which encode at least 170 amino acids at the C-termini. Thus, 31 sequences were from the TIGR database plus 7 more were produced in this study (i.e., newly identified genes named Glan and Barb [Genbank: AY880883-AY880888]). The maximum likelihood (ML) tree consisted of 3 single genes and 11 clades, containing 2 to 5 sequences (Figure 1). Seven clades (I-VII) could be regarded as a part of the multi gene family, as sequence identity with the nearest neighbors was in the range of 35–73%; the remaining 4 clades were highly divergent. Only 1 of 6 clades containing more than 3 genes (X in Figure 1) was split into clusters supported by high bootstrap values. The highest sequence identity were observed for Glan and Barb. However, divergence within other clades could in theory be overestimated, due to forced assembly of similar transcripts.

**Figure 2:**
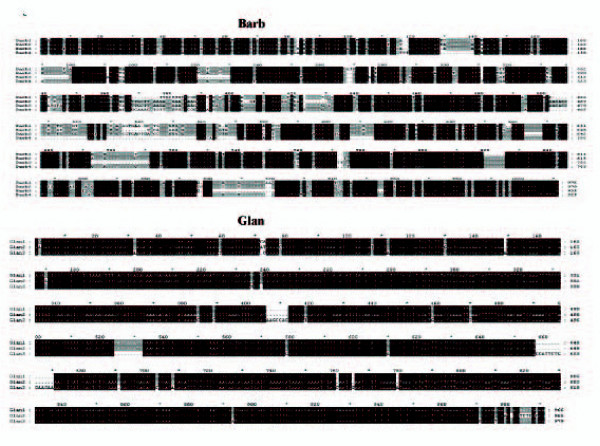
Alignment of protein coding sequences of new transcribed rainbow trout TC1-like transposases cloned in this study.

**Figure 3:**
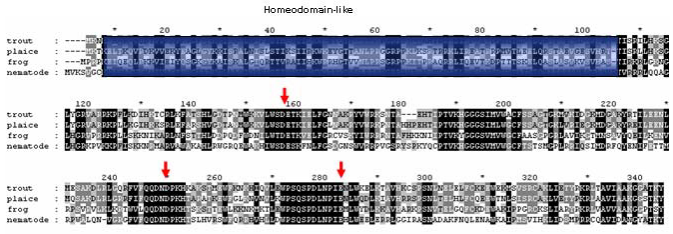
Alignment of deduced amino acid sequences of rainbow trout transposon Glan with the Tc1-like transposon of plaice, *Pleuronectes platessa *(Genbank: CAB51372), TPA of frog, *Rana pipiens *(Genbank: DAA01561) and tcb1 of the nematode, *C. elegans *(Genbank: NP_741053). Homeodomain (indicated with box) is involved in the binding of DNA; the DDE/D motif (indicated with arrows) is present in diverse MGE [1].

Sequencing of complete reading frames for 3 copies of Glan and 4 copies of Barb allowed for the study of transposons molecular evolution within the rainbow trout genome. All 7 sequences include incapacitating mutations, which prevent translation of transposase. Barb copies have diverged up to 11.4 ± 1.4% (mean ± SD) and accumulation of deletions (Figure 2) impeded reconstruction of the ancestral protein. Low divergence of Glan copies (1.9 ± 0.8%) suggest relatively recent transposition into the rainbow trout genome. The consensus sequence of 3 reading frames was identical to TIGR contig [TGI:TC46394], which encoded a protein with characteristic features of Tc1-like transposase, such as the presence of domains required for nuclear localization, DNA binding, cleavage and joining and DDE motif found in the catalytic units of diverse MGEs and retroviruses (Figure 3). Noteworthy of mention is that all transcripts of Glan contained mutations that prevented translation of transposase, however the consensus contig sequence that was assembled from a large number of EST from different cDNA libraries appeared intact. Given that the rate of spontaneous mutations in vertebrate germ cell lines is ~10^-5 ^[9], transposition of Glan could have taken place as recently as only a few thousand years ago. We also performed PCR screen of this gene in fish from inland reservoirs of Finland, where it was detected in 17 species from different orders (Table 2). Interestingly, three of the four species in which Glan was not found (grayling, whitefish and vendace) are more closely related to rainbow trout than most of those species carrying this gene. Low divergence of copies and discontinuous distribution are evidence for horizontal transmission. We analysed the rates of synonymous (Ks) and non-synonymous (Ka) substitutions in newly identified rainbow trout transposons using a sequence of the nearest Swissprot protein (hypothetical transposase of plaice, with 77% homology [Genbank:CAB51372]) as a reference (Table 3). With respect to this transposase, the Ks/Ka ratio was high and significantly greater in the younger gene (4.85 ± 0.30 in Glan and 3.35 ± 0.04 in Barb). A comparison of copies indicated a probability of divergent evolution in Glan (Ks/Ka = 0.69 ± 0.05). In Barb the rates of synonymous and non-synonymous substitutions approached unity (Ks/Ka = 1.03 ± 0.12), which is consistent with the protracted accumulation of mutations in a solely neutral mode.

**Table 2:** Presence of Glan in genomic DNA of fish from inland waters of Finland.

Species	Result
Arctic charr (*Salvelinus alpinus*)	Found
Brown trout (*Salmo trutta*)	Found
Smelt (*Osmerus eperlanus*)	Found
Grayling (*Thymallus thymallus*)	Not found
Vendace (*Coregonus albula*)	Not found
Whitefish (*Coregonus lavaretus*)	Not found
Pikepearch (*Sander lucioperca*)	Found
Perch (*Perca fluviatilis*)	Found
Ruffe (*Gymnocephalus cernuus*)	Found
Crucian carp (*Carassius carassius*)	Found
Roach (*Rutilus rutilus*)	Found
Bullhead (*Cottus gobio*)	Found
Bream (*Abramis brama*)	Found
Silver bream (*Abramis bjoerkna*)	Found
Bleak (*Alburnus alburnus*)	Found
Dace (*Leuciscus leuciscus*)	Found
Rudd (*Scardinius erythrophthalmus*)	Found
Ide (*Leuciscus idus*)	Found
Burbot (*Lota lota*)	Found
Northern pike (*Esox lucius*)	Found
Eel (*Anguilla anguilla*)	Found

**Table 3:** Synonymous (Ks) and non-synonymous (Ka) divergences of the rainbow trout transposons Glan and Barb. Plaice transposase was used as a reference.

Genes	Ks	Ka	Ks/Ka
*Glan*			

Rainbow trout (3 copies)	0.016 ± 0.007	0.022 ± 0.009	0.69 ± 0.05
Plaice transposase	0.69 ± 0.02	0.14 ± 0.01	4.85 ± 0.31

*Barb*			

Rainbow trout (4 copies)	0.12 ± 0.02	0.11 ± 0.01	0.99 ± 0.13
Plaice transposase	0.68 ± 0.02	0.20 ± 0.00	3.35 ± 0.04

**Figure 4:**
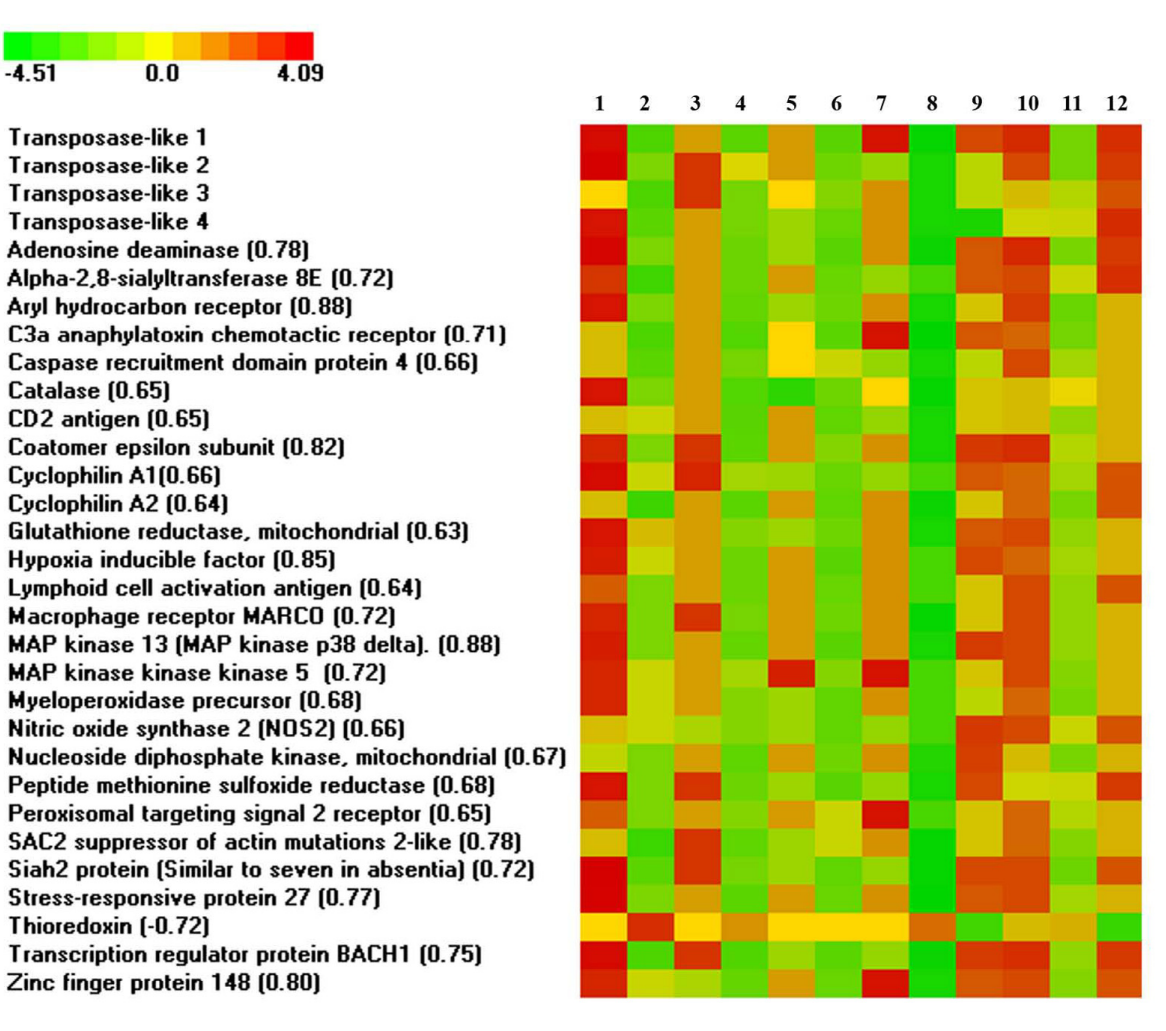
Differential expression of transposons in rainbow trout. The panel presents profiles of transposons and a group of genes that showed coordinated expression in 35 microarray experiments (Pearson r^2 ^is indicated). Selected experiments are reported: 1–8 – exposure of yolk sac rainbow trout fry to model contaminants [10], β-naphthoflavone, low (1) and high (2) doses; cadmium, low (3) and high (4) doses; carbon tetrachloride, low (5) and high (6) doses; pyrene, low (7) and high (8) doses. Items 9–12 – response to handling stress [11, GEO:GSM22355], kidney, 1 day (9) and 5 days (10); brain, 1 day (11) and 5 days (12).

We did not find sequences of any other known proteins in the salmonid Tc1-like contigs and probably transposons are transcribed from own promoters. Evidence for regulation of transposon transcription rate was produced in microarray analyses. We used a platform designed for studies of responses to environmental stress, toxicity and pathogens in salmonid fish [10,11]. Overall this platform included more than 1300 genes, 7 of which were similar to Tc1-like transposons. Five transposons showed marked differential expression in response to external stimuli, such as handling stress, exposure to toxic compounds and injection of cortisol or bacterial antigens; the microarray results were confirmed with real-time qPCR. A consensus profile of transposons correlated with those of 27 protein coding genes in 35 microarray experiments (Pearson r^2 ^> 0.63); examples are presented in Figure 4. The highest correlation (r^2 ^> 0.8) was shown by classical markers of cellular stress, such as the aryl hydrocarbon receptor, MAP kinase 13 and hypoxia inducible factor. We also searched for enrichment of Gene Ontology [12] categories in this list of genes. Significant over-representation was demonstrated by functional classes that are implicated in protective reactions to acute conditions (i.e., response to stress and oxidative stress, defense and humoral immune response, receptors and regulators of transcription, Table 4).

**Table 4:** Over-presentation of Gene Ontology classes in a list of genes that showed co-ordinated expression with Tc1-like transposons. The composition of microarray was used as a reference. The gene names and expression profiles are shown in Figure 4.

GO classes	P	Genes in list	Genes on chip
Antimicrobial humoral response	1 × 10^-5^	7	31
Defense response	2 × 10^-4^	12	102
Signal transduction	0,008	10	113
Cellular defense response	0,009	3	12
Response to oxidative stress	0,03	3	18
Transcription regulator activity	0,03	5	47
Receptor activity	0,04	5	49

^1^Exact Fisher's probability

## Discussion

Having a large number of transposons and a preponderance of Tc1-like genes is a characteristic feature of salmonid genomes [3-5]. Sequence analysis of the transcribed genes (Figure 1) suggested repeated transpositions at protracted intervals. A wide distribution of Tc1 transposons is believed to account for the limited requirements in the host cellular factors. Sleeping Beauty, an artificially reconstructed salmon transposon [13] is capable of integration into genomes of a wide range of vertebrate species, however different efficiencies observed in various cell lines point to possible involvement of the recipient's proteins in transposition [14]. This is in line with a wide, though limited, distribution of homologs for the transcribed salmonid DNA transposons, which have not been found among EST of warm-blood vertebrates. The variety of salmonid Tc1-like genes is truly remarkable. Phylogenetic analyses of 38 sequences, encoding homologous fragments of C-termini, found 14 distinct types of Tc1-like genes and the real number of different genes is probably much greater. Our search was based on the similarity between proteins that were available from Swissprot, and many transposons could remain unidentified due to the lack of known homologs. Furthermore, the rapid decay of transposons could impede the discovery of ancient transposed genes.

Despite the wide spread occurrence of Tc1-like transposons in vertebrates, not a single active gene has been identified to date [14]. Inactivation of salmonid DNA transposons could take place within a relatively short period of time after transmission. Cloning of 2 transposons having a relatively low divergence rate indicates the rapid accumulation of incapacitating mutations, such as insertions or deletions, shifts of reading frames and premature stop codons (Figure 2). Analysis of synonymous and non-synonymous substitutions suggest that inactivation of younger transposon could be preceded by selective divergence within a limited period of time, whilst evolution of the older gene appeared entirely neutral. Results from a study on recent transpositions in insects from four different orders suggest that selective constraints operate exclusively by horizontal gene transfer [15]. A comparison of rainbow trout genes with Tc1-like transposon from plaice confirm the conservation of functionally important domains in distantly related proteins, which is gradually obscured during the course of neutral evolution (K_s_/K_a _ratios in the younger Glan and older Barb genes are 4.85 ± 0.31 and 3.35 ± 0.04 respectively).

Silencing of transposons takes place at the transcriptional or post-transcriptional levels [16], and both of these mechanisms could act in salmonid fish. Based on frequency in a 1.2 MB gene fragment, we can assume that Tc1-like genes comprise nearly 5% of the Atlantic salmon genome and only a minor fraction preserved transcription after inactivation of translation. A survey of salmonid EST found untranslated transposons in both sense and anti-sense polarities, which is the main prerequisite for the formation of double-stranded RNA. RNA interference (RNAi) is implicated in the control of transposition in germ cell lines of the nematode *C. elegans *[17], and existence of an RNAi pathway in rainbow trout was recently demonstrated [18]. Suppression of intact transposases with mutant genes was also reported in insects, and this control mechanism is referred to as dominant-negative complementation [19].

Given efficient protection against transposition in animals, the tenacity and variety of transposons may seem surprising. Sustained persistence of transposons can, in theory, account for their residence in unknown reservoir species; e.g, the role of parasites as potential vectors of horizontal transfer across phylogenetically remote organisms has been hypothesized [20]. However this can hardly explain the remarkable diversity of these genes. The ML tree (Figure 1) suggests that at each transposition event, the rainbow trout genome was invaded with a new transposon, although several genes could have a common ancestor. If expression of translated genes is under control of RNAi, successful recurring transposition of identical or highly similar genes appears unlikely. Hence, the combination of neutral or divergent evolution within a genome with transfer across phylogenetic boundaries can be the most efficient strategy for the survival and diversification of transposons. PCR screen detected Glan in genomes of many fish species from phylogenetically remote taxonomic groups (Table 2). Clades I-VII of the ML tree (Figure 1) can correspond to genes that evolved independently. However it is also possible that descendants of a founder gene has returned several times into the rainbow trout genome, after passage through a chain of co-evolutionary hosts.

Results of our microarray studies suggested that a large fraction of transcribed Tc1-genes can be stimulated under acute conditions, but it remains unclear whether or not the transposon transcripts have any functional importance. In theory, they can be transcribed from cryptic promoters, which are activated by the remodelling of chromatin. However, input from stress-responsive promoters is also plausible. Transcripts can be required for the control of transposition through RNAi, however such explanation appears unlikely for highly mutated genes that were probably silenced long ago in evolutionary time. Currently, there is a growing body of evidence to support the involvement of non-coding RNA into the regulation of gene expression at different levels. The role of small and large RNA in modification of the chromatin structure was reviewed recently [21-23]. Stress-induced transcription of short interspersed repeated sequences (SINE) was reported in human, mouse and silkworm [24-27]; and SINE transcripts were shown to enhance translation of reporter genes [28,29]. Stress also activates the transcription of satellite III repeat [30]. Because this large non-coding RNA is consistently associated with chromatin, it can be required for the protection of sensitive regions from stress-induced damage. Synthetic double-stranded RNA enhances the expression of anti-viral proteins in salmonid fish [31,32] and, in theory, endogenous dsRNA can mimic a viral infection by launching protective reactions.

Tc1-like transposons are co-regulated with a group of genes that are implicated in the defense response, signal transduction and regulation of transcription. In this respect, it is noteworthy to mention that Tc1-like fragments reside in a number of immune and stress-related salmonid genes, such as the non-classical MHC class I antigen [Genbank:AF091779, Genbank:AF091780], immunoglobulin heavy chain, IgD [Genbank:AF141605, Genbank:AF278717], inducible nitric oxide synthase iNOS/NOS2 [Genbank:AJ295231] and aryl hydrocarbon receptor 2b, AhR2 [Genbank:AY463929]. Multiple copies of Glan in sense and anti-sense polarity are found in rainbow trout MHC class Ia [Genbank: AB162342.1] and b [AB162343.1] regions, in the vicinity of genes encoding the complement proteasome subunit and several MHCI loci. Modulation of gene expression that was due to the insertion of transposons has been documented in many studies (reviewed in [33]), and involvement of dispersed repeated sequences into the co-ordination of gene expression with similar functions was hypothesized more than three decades ago [34]. The role of transposon transcripts in the regulation of gene expression was recently discovered in yeast [35], where the induction of an RNAi-dependent silent chromatin configuration resulted in reduced transcription of several meiotic genes. A possible involvement of transposon transcripts in the regulation of gene expression in salmonid fish remains to be studied.

## Conclusion

Information produced by the sequencing of salmonid fish cDNA libraries and identification of recently transmitted transposons provide new insights into the structure, diversity and molecular evolution and life cycle of mobile genetic elements. High expression levels in rainbow trout tissues and marked responses to external stimuli indicate potential functional roles of transposon pseudogenes, which requires further investigation. These genes can be used as sensitive molecular biomarkers of acute conditions in salmonid fish.

## Methods

### Sequence analyses

The expressed transposons were analysed in rainbow trout and Atlantic salmon TIGR Gene indices, and sequence comparison was conduced with stand-alone blast [36]. Multiple sequence alignments were performed with ClustalW [37] and the conserved protein domains were searched in Interpro [38]. Synonymous and non-synonymous substitutions in newly cloned genes were determined by Dnasp [39]. Maximum Likelihood (ML) phylogenetic analyses were performed with Phylip [40].

### PCR cloning

The conserved sequence in the untranslated regions of rainbow trout Tc1-like transposases were inferred from EST sequences. RNA was extracted from rainbow trout brain and treated with Rnase-free Dnase (Promega). Reverse transcription with SuperScriptIII (Invitrogen) was primed with oligo(dT). PCR was performed with primer 5'-ATACAGTGCCTTGCGAGAGTATTC-3' using a TripleMaster kit (Eppendorf), and the product was cloned into pcDNA3.1/V5-His-TOPO (Invitrogen). Seven of nine sequenced clones contained complete reading frames.

### PCR analyses of genomic DNA

The fish samples were collected from inland reservoirs in Finland, and DNA from fin clips was prepared with salt extraction [41]. In brief, fin samples were digested at 60°C in 440 μl of buffer (1.8 mM EDTA, 9 mM Tris-HCl, pH 8; 1.8% SDS) containing 160 μg of proteinase K. After addition of 300 μl of 6 M NaCl, lysates were centrifuged at 12,000 *g *for 30 min. DNA in the supernatants was precipitated with isopropanol, washed with 70% aqueous ethanol and dissolved in water. The 654-base fragments of Glan PCR were amplified using the Hot Master Taq kit (Eppendorf). Primers (5'-TGAAGAATCGACAACAAGTGGGACA-3' and 5'-GCTTTCTTCTTGCCACTCTTCCATA-3') were annealed to templates at 68°C.

### Microarray analyses

Fish experiments, design of the rainbow trout cDNA microarray, hybridization protocol and data analyses are described in detail elsewhere [10,11]. In brief, the platform included 1,300 genes printed in 6 replicates each. The dye swap design was used; each sample containing RNA from 4 individuals was hybridized to slides with reverse assignment of fluorescent dyes (Cy3- and Cy5-dCTP from Amersham Pharmacia). Labels were incorporated at the stage of cDNA synthesis. The measurements in spots were filtered by criteria *I/B ≥ 3 *and *(I-B)/(S_I_*+*S_B_) ≥ 0.6*, where *I *and *B *were the mean signal and background intensities, respectively, and *S*_*I*, _*S*_*B *_were the standard deviations. Lowess normalization was performed and differential expression was analysed with the Student's t-test (p < 0.01). The genes were ranked by the log(p-level).

## Abbreviations

MGE – mobile genetic element; ML – maximum likelihood.

## Authors' contributions

AK carried out sequence analyses and drafted the manuscript, HK conducted the microarray analyses, SA performed the statistical analyses and HM coordinated research. All authors read and approved the final manuscript.

## References

[B1] Plasterk RH, Izsvak Z, Ivics Z (1999). Resident aliens: the Tc1/mariner superfamily of transposable elements. Trends Genet.

[B2] Robertson HM, Soto-Adames FN, Walden KKO, Avancini RMP, Lampe DJ, Syvanen M, Kado CI (1998). The mariner transposons of animals: horizontally jumping genes. Horizontal gene transfer.

[B3] Goodier JL, Davidson WS (1994). Tc1 transposon-like sequences are widely distributed in salmonids. J Mol Biol.

[B4] Radice AD, Bugaj B, Fitch DH, Emmons SW (1994). Widespread occurrence of the Tc1 transposon family: Tc1-like transposons from teleost fish. Mol Gen Genet.

[B5] Reed KM (1999). Tc1-Like Transposable Elements in the Genome of Lake Trout (Salvelinus namaycush). Mar Biotechnol.

[B6] Leaver MJ (2001). A family of Tc1-like transposons from the genomes of fishes and frogs: evidence for horizontal transmission. Gene.

[B7] Quackenbush J, Cho J, Lee D, Liang F, Holt I, Karamycheva S, Parvizi B, Pertea G, Sultana R, White J (2001). The TIGR Gene Indices: analysis of gene transcript sequences in highly sampled eukaryotic species. Nucleic Acids Res.

[B8] Pertea G, Huang X, Liang F, Antonescu V, Sultana R, Karamycheva S, Lee Y, White J, Cheung F, Parvizi B (2003). TIGR Gene Indices clustering tools (TGICL): a software system for fast clustering of large EST datasets. Bioinformatics.

[B9] Kohler SW, Provost GS, Fieck A, Kretz PL, Bullock WO, Sorge JA, Putman DL, Short JM (1991). Spectra of spontaneous and mutagen-induced mutations in the lacI gene in transgenic mice. Proc Natl Acad Sci USA.

[B10] Koskinen H, Pehkonen P, Vehniäinen E, Krasnov A, Rexroad C, Afanasyev S, Mölsä H, Oikari A (2004). Response of rainbow trout transcriptome to model chemical contaminants. Biochem Biophys Res Commun.

[B11] Krasnov A, Koskinen H, Pehkonen P, Rexroad CE, Afanasyev S, Mölsä H (2005). Gene expression in the brain and kidney of rainbow trout in response to handling stress. BMC Genomics.

[B12] Ashburner M, Ball CA, Blake JA, Botstein D, Butler H, Cherry JM, Davis AP, Dolinski K, Dwight SS, Eppig JT, Harris MA, Hill DP, Issel-Tarver L, Kasarskis A, Lewis S, Matese JC, Richardson JE, Ringwald M, Rubin GM, Sherlock G (2000). Gene ontology: tool for the unification of biology. The Gene Ontology Consortium. Nat Genet.

[B13] Ivics Z, Hackett PB, Plasterk RH, Izsvak Z (1997). Molecular reconstruction of Sleeping Beauty, a Tc1-like transposon from fish, and its transposition in human cells. Cell.

[B14] Izsvak Z, Ivics Z, Plasterk RH (2000). Sleeping Beauty, a wide host-range transposon vector for genetic transformation in vertebrates. J Mol Biol.

[B15] Lampe DJ, Witherspoon DJ, Soto-Adames FN, Robertson HM (2003). Recent horizontal transfer of mellifera subfamily mariner transposons into insect lineages representing four different orders shows that selection acts only during horizontal transfer. Mol Biol Evol.

[B16] Rudenko GN, Ono A, Walbot V (2003). Initiation of silencing of maize MuDR/Mu transposable elements. Plant J.

[B17] Sijen T, Plasterk RH (2003). Transposon silencing in the *Caenorhabditis elegans* germ line by natural RNAi. Nature.

[B18] Boonanuntanasarn S, Yoshizaki G, Takeuchi T (2003). Specific gene silencing using small interfering RNAs in fish embryos. Biochem Biophys Res Commun.

[B19] Hartl DL, Lohe AR, Lozovskaya ER (1997). Modern thoughts on an ancient marinere: function, evolution, regulation. Annu Rev Genet.

[B20] Kordis D, Gubensek F (1999). Molecular evolution of Bov-B LINEs in vertebrates. Gene.

[B21] Akhtar A (2003). Dosage compensation: an intertwined world of RNA and chromatin remodelling. Curr Opin Genet Dev.

[B22] Stevenson DS, Jarvis P (2003). Chromatin silencing: RNA in the driving seat. Curr Biol.

[B23] Wutz A (2003). RNAs templating chromatin structure for dosage compensation in animals. Bioessays.

[B24] Jang KL, Collins MK, Latchman DS (1992). The human immunodeficiency virus tat protein increases the transcription of human Alu repeated sequences by increasing the activity of the cellular transcription factor TFIIIC. J Acquired Immune Defic Syndr.

[B25] Fornace AJ, Mitchell JB (1986). Induction of B2 RNA polymerase III transcription by heat shock: enrichment for heat shock induced sequences in rodent cells by hybridization subtraction. Nucleic Acids Res.

[B26] Li T-H, Spearow J, Rubin CM, Schmid CW (1999). Physiological stresses increase mouse short interspersed element (SINE) RNA expression in vivo. Gene.

[B27] Kimura RH, Choudary PV, Schmid CW (1999). Silkworm Bm1 SINE RNA increases following cellular insults. Nucleic Acids Res.

[B28] Chu WM, Ballard R, Carpick BW, Williams BRG, Schmid CW (1998). Potential Alu Function: Regulation of the Activity of Double-Stranded RNA-Activated Kinase PKR. Mol Cell Biol.

[B29] Rubin CM, Kimura RH, Schmid CW (2002). Selective stimulation of translational expression by Alu RNA. Nucleic Acids Res.

[B30] Jolly C, Metz A, Govin J, Vigneron M, Turner BM, Khochbin S, Vourc'h C (2004). Stress-induced transcription of satellite III repeats. J Cell Biol.

[B31] Trobridge GD, Chiou PP, Kim CH, Leong JC (1997). Induction of the Mx protein of rainbow trout Oncorhynchus mykiss in vitro and in vivo with poly I:C dsRNA and infectious haematopoietic necrosis virus. Dis Aquat Org.

[B32] Jensen I, Larsen R, Robertsen B (2002). An antiviral state induced in Chinook salmon embryo cells (CHSE-24) by transfection with the double stranded RNA poly I: C. Fish Shellfish Immunol.

[B33] Labrador M, Corces VG (1997). Transposable element-host interactions: regulation of insertion and excision. Annu Rev Genet.

[B34] Britten RJ, Davidson EH (1969). Gene regulation for higher cells: a theory. Science.

[B35] Schramke V, Allshire R (2003). Hairpin RNAs and retrotransposon LTRs effect RNAi and chromatin-based gene silencing. Science.

[B36] Altschul SF, Madden TL, Schaffer AA, Zhang J, Zhang Z, Miller W, Lipman DJ (1997). Gapped BLAST and PSI-BLAST: a new generation of protein database search programs. Nucleic Acids Res.

[B37] Thompson JD, Higgins DG, Gibson TJ (1994). CLUSTAL W: improving the sensitivity of progressive multiple sequence alignment through sequence weighting, position-specific gap penalties and weight matrix choice. Nucleic Acids Res.

[B38] Mulder NJ, Apweiler R, Attwood TK, Bairoch A, Barrell D, Bateman A, Binns D, Biswas M, Bradley P, Bork P, Bucher P, Copley RR, Courcelle E, Das U, Durbin R, Falquet L, Fleischmann W, Griffiths-Jones S, Haft D, Harte N, Hulo N, Kahn D, Kanapin A, Krestyaninova M, Lopez R, Letunic I, Lonsdale D, Silventoinen V, Orchard SE, Pagni M, Peyruc D, Ponting CP, Selengut JD, Servant F, Sigrist CJA, Vaughan R, Zdobnov EM (2003). The InterPro Database, 2003 brings increased coverage and new features. Nucleic Acids Res.

[B39] Rozas J, Rozas R (1999). DnaSP version 3: an integrated program for molecular population genetics and molecular evolution analysis. Bioinformatics.

[B40] Felsenstein J (1989). PHYLIP – Phylogeny Inference Package (Version 3.2). Cladistics.

[B41] Aljanabi SM, Martinez I (1997). Universal and rapid salt-extraction of high quality genomic DNA for PCR-based techniques. Nucleic Acids Res.

